# Factors associated with mental health of young children during the COVID-19 pandemic in the Netherlands

**DOI:** 10.1186/s13034-023-00686-9

**Published:** 2023-12-13

**Authors:** L. J. G. Krijnen, W. M. van Eldik, T. T. M. Mooren, B. van Rooijen, P. A. Boelen, A. L. van Baar, M. Spuij, M. Verhoeven, M. R. Egberts

**Affiliations:** 1 Child and Adolescent Studies, Utrecht University, Utrecht, Netherlands; 2 Youz, Parnassia Psychiatric Institution, The Hague, The Netherlands; 3 Department of Clinical Psychology, Utrecht University, Utrecht, The Netherlands; 4 ARQ National Psychotrauma Centre, Diemen, The Netherlands; 5 TOPP-Zorg, Driebergen, The Netherlands; 6 Ingeborg Douwes Centrum, Centre for Psycho-Oncology, Amsterdam, The Netherlands

**Keywords:** COVID-19, Toddlers, Pre-schoolers, Early childhood, Mental health, Caregivers, Pandemic, Risk factors, Protective factors, Child well-being

## Abstract

**Background:**

The COVID-19 pandemic and accompanying societal measures have impacted children and their families all over the world. Little is known about the factors associated with mental health outcomes in young children (i.e., 1 to 6 years old) during the pandemic. The current study aimed to examine associations with potential risk and protective factors, i.e., direct COVID-19 exposure factors as well as within-family characteristics.

**Methods:**

Caregivers of children aged 1–6 years old were recruited in the Netherlands to participate in an ongoing longitudinal research project. In the current study, baseline data—collected during the 1st year of the pandemic—are reported. The final sample consisted of 2762 caregivers who answered questionnaires assessing negative and positive dimensions of their children’s mental health (i.e., anxiety, depressive symptoms, anger, sleep problems, positive affect, and self-regulation). Furthermore, caregivers provided information regarding: (1) Direct COVID-19 related factors, i.e., parental infection and death of a family member or close friend due to COVID-19, (2) Family related COVID-19 factors, i.e., parental perceived impact of the pandemic and COVID-19 related parent–child emotion regulation strategies (i.e., active, avoidant and information-focused strategies), (3) General caregiver’s distress, i.e., parental mental health, parental feelings of rejection towards their child. Regression analyses were used to examine associations with children’s mental health.

**Results:**

Direct COVID-19 related factors were not associated with more mental health problems in the children, though parental COVID-19 infections were related with less anger in children. Family related COVID-19 factors and caregiver’s distress were related with children’s mental health. Higher parental perceived negative impact of the pandemic, lower parental perceived positive impact of the pandemic, more avoidant as well as more active and information-focused parent–child emotion regulation strategies, more caregiver’s mental health problems and more parental feelings of rejection towards their child were related with more mental health problems in the child.

**Conclusion:**

Direct exposure to COVID-19 was not related with more mental health problems in the child. Family related COVID-19 factors and caregiver’s distress appear to play a more important role for young children’s mental health. Findings may inform prevention and intervention programs for potential future global crises as well as other stressful events.

**Supplementary Information:**

The online version contains supplementary material available at 10.1186/s13034-023-00686-9.

## Background

The COVID-19 pandemic, declared in March 2020 [1], has impacted children and their families worldwide. Since the start of the pandemic, measures were taken to restrict social contact, affecting families with children. Measures such as closure of day care centres and schools, forced parents to combine working from home while taking care of their children. Since the COVID-19 outbreak, increases in mental health problems among school-aged children, adolescents and adults have been reported globally [2–5]. Less is known regarding the mental health of infants and younger children (aged 1–6 years), despite the importance of this knowledge, as mental health problems in early childhood can increase the probability of psychopathology later in life [6, 7].

The few studies that focused on mental health of preschool aged children during the pandemic showed mixed results. Some studies highlighted resilience in young children and reported unchanged, or even improved, mental health from pre- to during pandemic [8, 9], whereas other studies identified increases in emotional problems during the pandemic, such as internalizing and externalizing problems, oppositional symptoms, sleeping problems and being upset by separation [10–14]. These mixed findings indicate that not all children were equally affected by the pandemic.

To understand these individual differences in young children, the current study investigated which factors are related with 1–6 years old children’s mental health during the pandemic, using a large community sample. Insight into which factors protect or negatively impact children’s mental health during a crisis like the COVID-19 pandemic may inform prevention and treatment programs to buffer young children against negative mental health outcomes not only in the context of the COVID‐19 pandemic and potential future crises, but also in other stressful circumstances for families with young children.

Three main categories of factors were studied: (1) Direct COVID-19 related factors, i.e., parental infection and death of a family member or close friend, (2) Family related COVID-19 factors, i.e., parental perceived impact of the pandemic and COVID-19 related parent–child emotion regulation strategies, (3) General caregiver’s distress, including parental mental health and parental feelings of rejection towards their child.

### Direct COVID-19 related factors

The experience with infection of a parent and death of a family member or friend during the pandemic may have impacted the mental health of young children negatively. Infections of COVID-19 within the family and perceived fear about infections were associated with more mental health problems in children and adolescents [15–17]. Furthermore, if a first-degree family member died from COVID-19, children suffered from more attention problems and pervasive developmental problems [17]. A loved one dying during the pandemic came with unique and serious challenges, such as isolation from family members including the dying member, less social support and increased fear in children to infect another family member [18].

### Family related COVID-19 factors

Parents’ perceptions on how the pandemic impacted their lives may have affected children’s mental health. Many parents perceived the impact of the societal measures regarding COVID-19—e.g., working from home while taking care of the children, financial stress, job loss, feelings of isolation—as negative [19–21]. This parent-reported negative impact of COVID-19 was related with children’s lower quality of life and increased loneliness [22]. Parents, however, also reported a positive impact of the pandemic, in terms of more family-time spent together and more appreciation of family relationships [23], which may buffer children’s mental health. Thus, while a negative perceived impact may form a risk factor for young children’s mental health, a positive perceived impact may form a protective factor against mental health problems.

How parents communicated with their children about the COVID-19 pandemic, referred to as parent–child emotion regulation strategies, can also affect children’s mental health. Parents may have used different emotion regulation strategies to help their children cope with the pandemic, by actively encouraging children to talk about their experiences and feelings (i.e., active parent–child emotion regulation), keeping information regarding COVID-19 away from their children (i.e., avoidant parent–child emotion regulation), or by using an information-focused strategy by explaining the pandemic, using child-friendly material (information-focused parent–child emotion regulation). Little is known yet regarding parent–child emotion regulation strategies during the pandemic and the current study will therefore explore the associations between these strategies and children’s mental health.

### General caregiver’s distress

Parental mental health problems and feelings of rejection towards their children have been related with children’s mental health in pre-pandemic circumstances [24–26]. Caregiving is already challenging under normative circumstances [27, 28], but the pandemic’s restrictions may have increased this challenge [29]. Indeed, maternal (parenting) stress, anxiety, and depression were found to be related with more externalizing and internalizing problems in young children during the pandemic (2–6 years old) [11, 30–32].

In addition, parental feelings of rejection, including both overt as well as more covert displays or feelings of disliking, dismissing or disapproving the child and its behavior, can increase during stressful circumstances and may affect children’s mental health [26, 33]. Parental rejection can also be expressed in subtle ways through showing indifference or indirect negative judgements, which are also known to be related with children’s mental health problems [26]. However, subtle forms of rejection towards the children have not been studied in relation to young children’s well-being in the context of the pandemic.

### Current study

In the current study, we examined the associations between these risk and protective factors and children’s mental health in a community sample of Dutch children (1–6 years) during the COVID-19 pandemic. It was expected that worse children’s mental health outcomes were associated with (1) more exposure to direct COVID-19 factors, i.e., infection of a parent and death of a family member or friend due to COVID-19, (2) higher parental perceived negative impact of the pandemic and less parental perceived positive impact, and (3) higher levels of general caregiver’s distress—i.e., more parental mental health problems and more feelings of rejection towards the child. Associations between the three parent–child emotion regulation strategies and children’s mental health outcomes were examined exploratory as no expectation could be formulated based on the limited previous studies.

## Methods

### Design

The COVID-19 Unmasked project concerns a prospective longitudinal cohort study in which caregivers filled out an online survey at four time-points, i.e., at baseline, 3, 6 and 12 months later. It is part of a larger global study which was conceptualized in Australia and is conducted in multiple countries (for more information see [34]). The current study reports on the baseline data in the Netherlands, collected between November 2020 and June 2021. When recruitment started, the Netherlands were in a partial lockdown, which was followed by a hard lockdown (including day care- and school closures) on December 14th, 2020. Day care and schools re-opened on February 8th, 2021. At the end of January 2021, a curfew was introduced, which lasted until the end of April 2021. In June 2021, many of the COVID-19 restrictions were suspended, only to be reinstalled again—after collection of the baseline data—in November 2021.

### Participants

Parents living in the Netherlands with a young child were recruited using the following inclusion criteria: (1) being a caregiver of ≥ 18 years old, (2) taking care of a child aged between 1 and 6 years old, i.e., ≥ 11 months up till ≤ 72 months, (3) having access to internet to complete the survey, and (4) having sufficient knowledge of either Dutch or English as the survey could be completed in both languages.

### Procedure

Participants were recruited using convenience sampling and snowballing. Digital flyers were distributed through social media, day care centers, primary schools, the Dutch municipal health service, the website of Utrecht University, and the authors’ personal and professional networks. In addition, after some time, an online campaign was used targeting fathers and caregivers with a migration background as these subgroups were underrepresented in the sample at that time. On the first page of the survey, an information letter was shown followed by an informed consent form. After providing active consent, the participant could start the survey, which took approximately 20–45 min to complete. Caregivers willing to participate in the follow-up surveys shared their e-mail address. Participants could stop at any point in time and could leave questions open if they preferred not to answer. Ethical approval was provided by the Ethics Committee of the Faculty of Social and Behavioural Sciences of Utrecht University (20-408).

### Measures

#### Children’s mental health

The Patient-Reported Outcomes Measurement Information System Early Childhood (PROMIS EC; [35]) was used to assess children’s mental health. The PROMIS EC is a caregiver-report questionnaire, measuring four overall health domains, i.e., global, mental, social and physical health of children aged one to five. These domains also had specific dimensions. In the current study, we considered the scores on five dimensions, namely (i) Anger/Irritability (four item short form, e.g., “My child had temper tantrums”, α = 0.83), (ii) Anxiety (eight item short form, e.g., “My child seemed worried”, α = 0.87), (iii) Depressive symptoms (four item short-form, e.g., “My child was withdrawn”, α = 0.83), (iv) Sleep Problems (four item short form, e.g., “My child had difficulty falling asleep”, α = 0.84), (v) Self-regulation—Frustration Tolerance (six items, e.g., “My child could wait if asked, even if he/she really wanted to do something”, α = 0.89), and (vi) Positive affect (four item short form, e.g., “My child was happy”, α = 0.85). All items were answered on a 5-point Likert scale (1 = *never* to 5 = *always*). For every dimension, item scores were summed, leading to sum scores ranging from 4 to 20 for the four item dimensions, and 6 to 30 and 8 to 40 for the six item and eight item dimensions respectively. Hence, T-scores ranging from 0 to 100 were calculated, with a mean score of 50 and one standard deviation of 10 (i.e., based on reference tables from a pre-pandemic USA sample; see [36]). Additionally, T-scores were recoded into summary scores to indicate whether a child scored within the average range (± < 1 SD from the mean), scored moderately below/above the average mean (± 1.0–2.0 SD from the mean) or very low or very high (± > 2SD from the mean). For the current study, the PROMIS EC was translated to Dutch in collaboration with the Dutch-Flemish PROMIS National Center. The translation procedure included forward translation, back translation and cognitive debriefing with five caregivers. Good psychometric properties have been reported for the PROMIS EC in a USA sample [35, 37–41].

#### Direct COVID-19 exposure

##### Exposure to COVID-19 infections and death

The COVID-19 Pandemic Exposure and Loss Questions (PELQ; [34]) is a self-report questionnaire of 14 items which has been developed for the COVID-19 Unmasked study to assess direct effects of exposure and loss by children and their family members during the COVID-19 pandemic. For the purpose of the current study, we used information from three items to create two variables representing whether one of the caregivers had been infected with COVID-19. The first item asked “Were you ever suspected to have COVID-19?”, and when answered ‘yes’ participants could choose from the following answers: 1 = *Yes, was suspected but was not tested*, 2 = *Yes, I was tested for COVID-19 but did not have it*, 3 = *Yes, I was tested and diagnosed with COVID-19 and treated in isolation at home*, and 4 = *Yes, I was tested and diagnosed with COVID-19 and treated in isolation at hospital*. The second item asked: “Was another family member or person close to your child diagnosed with COVID-19?” (0 = *no*, 1 = *yes*). If answered with yes, participants had to choose the relationship of the child of that person (1 = *parent*, 2 = *sibling*, 3 = *grandparent*, 4 = *other*). If participants chose answer 3 or 4 for the first question and/or answer 1 for the second question, they were assigned a 1 to our variable ‘COVID-19 infection’ which was defined as ‘at least one caregiver had been infected with COVID-19’ (0 = none of the caregivers had been infected with COVID-19). Whether families had lost a family member or close friend of the family due to COVID-19 was assessed with one item, “A family member or close friend died from COVID-19” (0 = *no*, 1 = *yes*). If participants answered ‘yes’, a follow-up question was asked to specify the loss (1 = *parent of the child*, 2 = *sibling of the child*, 3 = *grandparent of the child*, 4 = *other*).

#### Family related COVID-19 factors

##### Perceived impact of the COVID-19 pandemic

The Pandemic Impact Questionnaire: Early Childhood (PIC-EC; [34]) was developed for the COVID-19 Unmasked study to measure the negative perceived impact (15 items, α = 0.82) and positive perceived impact (seven items, α = 0.77) of the COVID-19 pandemic on family life using self-report. The perceived negative impact items were used to measure how disruptions on daily routines, lifestyle and activities were experienced by the family, the caregiver and the child (e.g., “I have experienced increased tension or disconnection with my child/ren” and “My child has been affected by missing events important to them (e.g., their birthday party)”. The positive impact scale is based on five domains of posttraumatic growth, which include “new possibilities”, “relating to others”, “spiritual change”, “personal strength” and “appreciation of life” (e.g., “More quality family time”) [42]. All items are answered on a 5-point Likert scale ranging from 0 (*not at all*) to 4 (*very much*). Sum scores for both the positive and negative impact scale were calculated. Sum scores for the negative perceived impact could range from 0 to 60 and for the positive perceived impact from 0 to 28.

##### Parent–child emotion regulation strategies

The COVID-19 Pandemic Parenting Survey (CPPS; [34]) was developed for the COVID-19 Unmasked study to assess caregivers’ strategies to communicate with and inform their children about the COVID-19 pandemic. The CPPS is a self-report measure and consists of 14 items with answer options ranging from 0 (*not at all*) to 4 (*a lot*). Items asked how often caregivers talked with their child about the pandemic and the child’s thoughts and feelings, positively reframed emotional and cognitive experiences of the child, avoided talking about COVID-19 related topics or used child-friendly informative material. An exploratory factor analysis was carried out on the current sample using SPSS and revealed three factors, representing three emotion regulation strategies that parents can use to help their child cope with the pandemic: (1) *active parent–child emotion regulation* (six items, e.g., “I encourage my child to talk about their thoughts and feelings”) (α = 0.71), (2) *avoidant parent–child emotion regulation* (four items, e.g., “I try to avoid my child seeing or hearing any information about COVID-19) (α = 0.67), (3) *information-focused parent–child emotion regulation* (one item, i.e., “I’ve provided my child with child-friendly materials about COVID-19 to help them understand”). Sum scores for the active and avoidant emotion regulation strategies were calculated, which could range from 0 to 24 and 0 to 16 for the active and avoidant strategies respectively.

#### Caregiver’s distress

##### Caregiver’s mental health

The Depression, Anxiety and Stress Scale (DASS-21; [43, 44]) is a self-report questionnaire measuring depressive, anxiety and stress symptoms over the past week. It has been derived from the longer DASS-42 version [45]. The DASS-21 contains 21 items, with every subscale containing seven items (Stress: e.g., “I found it hard to wind down”, Anxiety: e.g., “I felt I was close to panic”, Depression: e.g., “I felt down-hearted and blue”), with answer options ranging from 0 (*Did not apply to me at all*) to 3 (*Applied to me very much or most of the time*). A total DASS-21 score was calculated by adding the subscales’ sum scores (α = 0.93), in which sum scores could range from 0 to 63. Psychometric properties of the Dutch DASS-21 have been examined in a large non-clinical sample, demonstrating good psychometric qualities [46].

##### Parental feelings of rejection towards child

The Parenting as Social Construct Questionnaire – Toddler version (PSCQ–T) [47] is a self-report questionnaire regarding parenting behavior. Initially, two subscales were selected for the COVID-19 Unmasked study: *warmth* (four items, e.g., “I can always find time for my child”) and *rejection* (four items, e.g., “Sometimes my child is hard to like”). Because the subscales showed low internal consistencies in the Australian sample, we examined the reliability of both scales in the current sample. Only the rejection scale appeared to be reliable (α = 0.60) and confirmed by factor analysis, and was therefore the only subscale used in the current study. Items were answered on a 4-point Likert scale ranging from 1 = *not true at all* to 4 = *very true*. Reliability and validity have previously proven to be acceptable [47]. The four items in this scale were: “I don’t understand my child very well”, “Sometimes my child is hard to like”, “At times the demands that my child makes feel like a burden”, “My child needs me more than I have time to give him/her”. These items seem to represent more subtle, covert feelings of rejection that parents can have towards their child which is why we refer to this subscale as parental *feelings* of rejection. A sum score was calculated, which could range from 4 to 24.

#### Demographic characteristics

In the first and last part of the survey, sociodemographic characteristics of the child, caregivers, and family were collected regarding sex, age, relationship status, previous health conditions, education level, job, and country of birth.

### Statistical analyses

All analyses were conducted in SPSS 26.0. Missing data on the PROMIC EC, PIC-EC, CPPS, DASS-21, and PSCQ-T were handled using mean imputation on the subscale level, but only when no more than 50% per subscale was missing. First, descriptive statistics, including bivariate Pearson correlations (continuous variables) and Point biserial or Phi correlations (categorical variables) were calculated. Then, to describe the level of negative and positive child mental health symptoms, summary scores on the six PROMIS EC subscales (i.e., anxiety, depression, anger, sleep problems, positive affect and self-regulation) were calculated. As the societal measures differed over time, we checked whether the date of filling out the questionnaires affected the answers on the measurements. A dichotomous variable was created: 0 = hard-lockdown (i.e., day care centers and schools were closed, which was from December 14th 2020 until February 8th 2021), 1 = (re-)opening (day care centers and schools were open, which was before December 14th 2020, and after February 8th 2021). Hence, independent samples t-tests were executed to test whether the descriptive statistics of the predicting variables and children’s mental health outcomes differed between these two time points. Finally, to examine associations of the risk and protective factors with child mental health outcomes, six regression analyses were conducted separately for the six mental health outcomes variables. As predictors, we included six variables: two direct COVID-19 variables—i.e., infection of at least one of the parents, and death of a family member or close friend due to COVID-19—, two family related COVID-19 factors—i.e., parental perceived impact of the pandemic and COVID-19 related parent–child emotion regulation strategies (i.e., active, avoidant, and information-focused emotion regulation strategies)—and two caregiver’s distress variables—i.e., caregiver’s mental health and parental feelings of rejection. We included the following covariates: the age and sex of the child and the sex and relationship status of the caregiver. All independent variables were added simultaneously to the model, similarly for all six models.

## Results

### Participants

A total number of 4016 participants gave their informed consent. Participants were included in the current study when at least one of the six children’s mental health outcomes was filled out, leading to an exclusion of 31% of the participants, resulting in a final sample of 2762 participants. Table 1 displays the participants’ characteristics.

**Table 1:** Participants’ characteristics

N = 2762	Children	Caregivers
Sex		
Female	1343 (48.6%)	2648 (95.9%)
Male	1408 (51.0%)	109 (3.9%)
Other/prefer not to say	11 (0.4%)	5 (0.2%)
Age in months (child) or years (caregiver)		
Mean (SD)	43.29 (16.25)	34.66 (4.09)
Range	11–72	22–50
Relationship status^a^		
Together	–	1875 (67.9%)
Single	–	67 (2.4%)
Educational level^b^		
Low	–	5 (0.2%)
Medium	–	463 (16.8%)
High	–	1473 (53.3%)
Country of birth^c^		
The Netherlands	2740 (99.2%)	1888 (68.4%)
Other	22 (0.8%)	59 (2.1%)
Relationship		
Biological caregiver	–	2738 (99.1%)
Other	–	20 (0.9%)
Previous health problems		
No	2526 (91.5%)	1453 (52.6%)
Yes^d^	224 (8.1%)	485 (17.6%)
At high risk due to chronical condition,		
Yes (%)^e^	32 (1.2%)	333 (12.1%)
Essential job^f^	–	1420 (51.5%)

^a^Together indicated being married, registered partnership, in a relationship. Alone referred to single, divorced, widow. Information of 820 participants was missing (29.7%)

^b^Educational level is divided into *low* referring to only primary school; *medium* referring to high school and secondary vocational education; and *high* referring to university or university of applied sciences. Information was missing for 821 participants (29.7%)

^c^Information regarding country of birth from the caregivers was missing for 815 caregivers (29.5%), but for none of the children

^d^Problems in children consisted of emotional or psychological problems, developmental disorders, sleeping-, eating- or crying problems, caregiver-child relationship difficulties, sensory processing problems, physical handicaps, chronic health issues, other issues that the caregiver considered as previous health condition. For 12 participants, data were missing. Problems in adults consisted of emotional problems such as burn-out symptoms, fatigue, (post-natal) depression, Attention Deficit Hyperactivity Disorder, Autism Spectrum Disorder, post-traumatic stress symptoms, substance use problems. Information was missing for 824 adults (29.8%)

^e^Percentage of children and caregivers with a chronic condition making them at high risk for negative outcomes after a COVID-19 infection

^f^Caregivers with an essential or ‘frontline’ occupation, such as jobs in health care, transport, education, day care. Data of 826 caregivers were missing (29.9%)

Participants that did not complete the questionnaire (n = 2114, 52.6%) did not differ from participants that completed the questionnaire (n = 1902, 47.4%) on child’s and caregiver’s sex (*p’*s > 0.22). A difference of approximately 1 month was observed for children’s age (mean_completers_ = 44.35, SD_completers_ = 16.25; mean_non-completers_ = 43.23, SD_non-completers_ = 17.48, *p* < 0.05).

### Descriptive statistics

Samples sizes, means, standard deviations and correlations among study variables are reported in Additional file 1: Table S1. The correlations between the six different children’s mental health outcomes were of weak to moderate strength, indicating that distinct concepts were measured that each can have a unique relationship with the risk and protective factors. Descriptive results regarding the direct COVID-19 exposure variables revealed that death of a friend or family member due to COVID-19 was reported 152 times (5.5%), of which one participant reported that the child’s parent died (< 1%), 34 parents reported that the child’s grandparent died (1.2%), and 117 caregivers answered ‘other’ (4.2%), indicating that a good friend or a less close family member (e.g., cousin or aunt) of the family died.

Results on the PROMIS EC showed that most children (i.e., > 80.0%) fell within the normal range on anger, anxiety, depressive symptoms and sleeping problems, and showed average to high scores on self-regulation and positive affect. See Table 2 for the profiles of mental health scores on the PROMIS EC.

**Table 2:** Profiles of the mental health scores (PROMIS EC) of young children during the COVID-19 pandemic

Anger (*N* = 2762)^a^	
Normal range	2471 (89.5%)
Moderate	269 (9.7%)
Severe	22 (0.8%)
Anxiety (*N* = 2637)^a^	
Normal range	2277 (86.3%)
Moderate	323 (12.2%)
Severe	37 (1.4%)
Depressive symptoms (*N* = 2508)^a^	
Normal range	2006 (80.0%)
Moderate	483 (19.3%)
Severe	19 (0.8%)
Sleeping problems (*N* = 2315)^a^	
Normal range	1970 (85.1%)
Moderate	315 (13.6%)
Severe	30 (1.3%)
Positive affect (*N* = 2415)^b^	
Low	430 (15.4%)
Average	1767 (73.2%)
High	218 (9.0%)
Self-regulation (*N* = 2413)^b^	
Low	371 (15.4%)
Average	1827 (75.7%)
High	215 (8.9%)

Data were missing for anxiety (*N* = 125), depression (*N* = 254), sleep (*N* = 447), positive affect (*N* = 347), and self-regulation (*N* = 349)

^a^Normal range refers to < 1 SD above the mean, moderate refers to 1–2 SD above the mean, severe refers to ≥ 2SD above the mean

^b^Low refers to > 1 SD below the mean, and high refers to > 1 SD above the mean

The predicting variables—i.e., direct COVID-19 factors, family related COVID-19 factors and caregiver’s distress—as well as children’s mental health outcomes were not significantly different between the caregivers that filled out the measurements of the current study during the hard-lock down measures, compared to the caregivers that answered the measurements during the (re-)opening period (all *p*’s > 0.05).

### Associations with children’s mental health

See Table 3, for an overview of the regression analyses results. Results are presented per regression analysis, i.e., per child mental health outcome. All effects were small, i.e., beta < 0.30 (Cohen, 2013), with the exception of a moderate effect size for parental feelings of rejection towards their child on self-regulation of the child.

**Table 3:** Results of the regression analyses for the six mental health outcomes

	Anger	Anxiety	Depression	Sleep	Positive affect	Self-regulation
Child age	< 0.01	0.03	0.05*	− 0.21***	− 0.08***	0.04*
Child sex	0.09***	− 0.04*	< .01	− 0.03	< 0.01	− 0.05*
Caregiver sex	< − 0.01	− 0.02	< − 0.01	− 0.03	− 0.03	0.06**
Relationship status	− 0.05*	− 0.03	− 0.03	− 0.02	< 0.01	0.02
*Direct COVID-19 exposure*						
COVID-19 infection	− 0.04*	− 0.02	− 0.02	0.01	< − 0.01	0.02
Death due to COVID-19	0.02	0.03	< 0.01	0.04	0.03	-0.02
*Family related COVID-19 factors*						
Negative COVID-19 impact	0.18***	0.24***	0.24***	0.15***	-0.21***	-0.17***
Positive COVID-19 impact	− 0.08***	0.01	− 0.02	− 0.07**	0.15***	0.12***
Active emotion regulation	0.13***	0.09***	0.07***	0.09***	− 0.01	− 0.03
Avoidant emotion regulation	0.03	0.08***	0.06**	0.02	− 0.04	0.01
Information-focused emotion regulation	0.05*	0.11***	0.05*	0.08***	− 0.04	< -0.01
*Caregiver’s distress*						
Caregiver’s mental health problems	0.09***	0.14***	0.15***	0.13***	− 0.03	− 0.09***
Feelings of rejection	0.28***	0.10***	0.16***	0.09***	− 0.29***	− 0.35***
R^2^	0.27	0.24	0.25	0.14	0.27	0.29
F	55.33***	46.87***	49.43***	25.02***	54.28***	60.24***

Standardized regression coefficients (i.e., beta) are presented. Child and caregiver sex was operationalized with 0 = ‘female’ and 1 = ‘male/other’. Relationship status: 0 = ‘not in a relationship’ and 1 = ‘in a relationship’. Caregiver COVID-19 infection: 0 = ‘no’, 1 = ‘yes’. Death due to COVID: 0 = ‘no’, 1 = ‘yes’. Sleep = sleeping problems

#### Children’s anger

The total model explained a significant proportion, i.e., 27%, of the variance in child’s anger (*R*^2^ = 0.27, *F*(13, 1918) = 55.33, *p* < 0.001). Two of the four covariates, i.e., child’s sex and relationship status of the parent, showed significant associations with children’s anger. First, caregivers reported higher anger levels in boys than in girls. Furthermore, single caregivers reported higher levels of anger problems in their children than caregivers in a relationship. Regarding the direct COVID-19 variables, infection of at least one of the parents predicted lower levels of anger problems in the child. Death of a family member or friend did not predict children’s anger levels. Regarding the family related COVID-19 variables, higher levels of experienced negative COVID-19 impact by the parents was associated with higher levels of children’s anger, whereas caregivers who experienced a greater positive impact reported relatively lower levels of anger problems. Higher levels of parental active and information-focused emotion regulation strategies were associated with higher levels of anger in the children. The use of the avoidant strategy was not associated with anger problems. Regarding caregiver’s distress variables, more mental health problems in caregivers were related with more anger in their children. Finally, more feelings of rejection towards their child were associated with higher levels of children’s anger.

#### Children’s anxiety

The total model explained a significant proportion, i.e., 24%, of the variance in child’s anxiety (*R*^2^ = 0.24, *F* (13, 1918) = 46.87, *p* < 0.001). Regarding the covariates, caregivers reported higher levels of anxiety in girls than boys. None of the other covariates formed significant associations with children’s anxiety. None of the COVID-19 exposure variables were associated with the level of anxiety in children. Of the family related COVID-19 variables, caregivers who reported a greater negative impact of COVID-19, reported higher levels of children’s anxiety. The perceived positive impact of COVID-19 was not related with anxiety levels of the children. The use of active, avoidant and information-focused emotion regulation strategies were all related with higher levels of children’s anxiety. Of the caregiver’s distress variables, results showed that caregivers who experienced more mental health problems and more feelings of rejection towards their child, reported higher levels of anxiety in their child.

#### Children’s depressive symptoms

The total model explained a significant proportion, i.e., 25%, of the variance in child’s depressive symptoms (*R*^2^ = 0.25, *F* (13, 1918) = 47.75, *p* < 0.001). One of the covariates, i.e., child’s age, showed a significant association with children’s depressive symptoms: Caregivers of older children reported higher levels of children’s depressive symptoms. None of the COVID-19 exposure variables were associated with children’s depressive symptoms. Regarding the family related COVID-19 variables, caregivers who reported a greater negative impact of the pandemic reported relatively higher levels of children’s depressive symptoms, whereas the experienced positive impact showed no association with this outcome. Parental use of active, avoidant, and information-focused emotion regulation strategies were all associated with higher levels of children’s depressive symptoms. Caregiver’s distress, in terms of more parental mental health problems and more feelings of rejection towards their child, was related with higher levels of depression in their child.

#### Children’s sleep problems

The total model explained a significant proportion, i.e., 14%, of the variance in child’s sleep problems (*R*^2^ = 0.14, *F* (13, 1918) = 25.02, *p* < 0.001). One covariate, i.e., child’s age, formed a significant association with children’s sleep problems: parents reported lower levels of sleep problems for older children than for younger children. None of the COVID-19 exposure variables were associated with sleep problems. Next, regarding the family related COVID-19 variables, caregivers who experienced a greater negative impact reported relatively higher levels of sleep problems, whereas caregivers who experienced a greater positive impact reported relatively lower levels of sleep problems. The use of active and the use of information-focused emotion regulation strategies were both associated with higher levels of children’s sleep problems. No effect of the avoidant strategy was found. Regarding caregiver’s distress, more parental mental health problems and more parental feelings of rejection towards their child were related with more sleep problems of their child.

#### Children’s positive affect

The total model explained a significant proportion, i.e., 27%, of the variance in child’s positive affect (*R*^2^ = 0.27, *F* (13, 1918) = 54.28, *p* < 0.001). One covariate, i.e., child’s age, showed a significant association with children’s positive affect: Caregivers of older children reported lower levels of positive affect in their child. None of the direct COVID-19 exposure variables were associated with children’s positive affect. Of the family related COVID-19 variables, caregivers who experienced a greater negative impact, reported lower levels of positive affect in their child, whereas caregivers who experienced a greater positive impact reported relatively higher levels of positive affect. None of the emotion regulation strategies used by caregivers were associated with children’s positive affect. Regarding caregiver’s distress, caregiver’s mental health was not associated with the positive affect of children, but more feelings of rejection towards their child were associated with lower levels of positive affect in children.

#### Children’s self-regulation

The total model explained a significant proportion, i.e., 29%, of the variance in child’s self-regulation (*R*^2^ = 0.29, *F* (13, 1918) = 54.28, *p* < 0.001). Three of the four covariates showed significant associations with children’s self-regulation: Older children and girls showed higher levels of self-regulation than younger children and boys. Moreover, fathers reported higher levels of self-regulation than mothers. The direct COVID-19 exposure variables were not associated with children’s self-regulation. Of the family related COVID-19 variables, caregivers who experienced a greater negative impact reported lower levels of children’s self-regulation, whereas caregivers who experienced a greater positive impact reported relatively higher levels of self-regulation in their children. None of the emotion regulation strategies were associated with children’s self-regulation. Regarding caregiver’s distress, caregivers who experienced higher levels of mental health problems themselves and reported more feelings of rejection towards their child, reported lower levels of self-regulation for their child.

## Discussion

In a large community sample, associations between young Dutch children’s mental health and potential risk and protective factors during the COVID-19 pandemic were studied. Our results showed that direct COVID-19 related factors were generally not related with more mental health problems in the children. Family related COVID-19 factors and caregiver’s distress were consistently related with children’s mental health: Higher parental perceived negative impact of the pandemic, lower perceived positive impact of the pandemic, more avoidant as well as more active and information-focused parent–child emotion regulation strategies, higher levels of caregiver’s mental health problems and feelings of rejection towards their child were all related with more mental health problems in the children. Effect sizes were relatively small. In conclusion, children’s mental health during the pandemic was found to be especially associated with factors within the family, rather than to direct COVID-19 related factors.

Focusing on children’s mental health during the COVID-19 pandemic, our results indicate that more than 80% of the caregivers reported a normal level of anger (e.g., frustration, tantrums), anxiety (e.g., fearfulness, social anxious behaviors), depressive symptoms (e.g., withdrawn, sad), and sleep problems (e.g., delated sleep, sleep discontinuity) in their children, according to the USA norms for the PROMIS EC [36]. Similarly, most caregivers (> 80%) reported average to high levels of positive affect and self-regulation, indicating that their child was often happy and playful and able to manage frustrations and stay calm when faced with a challenge. Of all the mental health outcomes, depressive symptoms were mostly reported: 19% of the caregivers observed moderate symptoms in their child. In addition, some age and sex differences were found: girls showed slightly higher levels of anxiety and self-regulation than boys. Caregivers of older children observed more depressive symptoms and less positive affect, but also better self-regulation and less sleep problems. These age effects might be explained from a developmental perspective, reflecting that older children might have developed more consistent sleep patterns and more self-regulation strategies and a greater display of different emotions.

In contrast to our hypothesis, a COVID-19 infection of a parent or death of a family member or friend was not associated with more mental health problems in the children. These findings were inconsistent with findings for older children, that showed more negative mental health outcomes when family members were infected or died [15–17]. An explanation for our results may be that young children are less aware of COVID-19 infections of a caregiver and death outside of the direct household. In the current study, the majority of the reported deaths consisted of a deceased friend of the family or a family member outside of the household. Therefore, the children may not have noticed substantial changes in their daily lives. With respect to the impact of COVID-19 infections of the parents, there was one exception in our results: Children of caregivers who had been infected with COVID-19 showed less anger than children of caregivers who had not been infected. This may be explained in at least two ways. Levels of stress in families might have decreased once infected people recovered from COVID-19, compared to the continuous fear of getting infected and the unknown consequences. Alternatively, this finding may be explained by the dynamics observed in families confronted with severe caregiver illnesses, in which children want to protect their parents from further distress (e.g., [48]). Similarly, young children may not have wanted to burden their caregivers when they were ill, or shortly after experiencing that their parents were threatened by the COVID-19 virus, and may therefore have adjusted their behavior accordingly.

Family related COVID-19 factors (i.e., parental negative and positive perceived impact of the pandemic and parent–child emotion regulation strategies) were related with mental health of the children. As hypothesized, children from parents who perceived a greater negative impact and/or a lower positive impact of the pandemic on their daily lives, showed higher levels of anger, anxiety, depressive symptoms and sleep problems as well as lower levels of positive affect and self-regulation. These findings align with studies examining families with children from a broader age range [19, 20, 22]. This may be explained by parental behaviors towards the child; if the parent had a negative perception of the pandemic, the parent may have—either directly or indirectly—expressed negative thoughts or feelings regarding the pandemic towards the child, which in turn impacted the child’s view on the pandemic and their well-being. However, it is important to realize that the associations can work both ways. More mental health problems in children could have increased parents’ negative perception of the pandemic, given the added challenges of caring for a child with mental health issues amidst limited support during the crisis. More research is needed to study the direction of the associations found in the current study. Furthermore, it is important to note that an independent association was found for negative perceived impact with child outcomes as well as for positive perceived impact and child outcomes. Positive perceived impact, such as increased family closeness and connection and new possibilities and appreciation of life were related with better child wellbeing in terms of more positive affect and more self-regulation and less anger and sleeping problems. This suggests that the negative and positive impact of the pandemic are not simply part of one underlying dimension and each have a unique relationship with the child’s wellbeing or with the caregiver’s perception of the child’s mental health.

The second family related COVID-19 factor considered in relation to children’s mental health was parent–child emotion regulation strategies. In the current study, greater use of all three emotion regulation strategies, i.e., active, avoidant and information-focused, was consistently associated with higher levels of mental health problems in young children. This is remarkable, because the active and avoidant strategies can be regarded as opposite strategies. The active strategy can generally be interpreted as an adaptive strategy to co-regulate emotions and cope with stressful circumstances, whereas avoidant strategies are generally considered maladaptive [49]. Furthermore, in light of a study showing that young children are likely to have misconceptions of causality and risk [50], one could have expected a positive effect of information-focused strategies. However, it is important to note that due to the cross-sectional design of the current study, the direction of effects between emotion regulation strategies and children’s well-being could not be tested. It is therefore possible that higher levels of mental health problems in the children have evoked more emotion regulation strategies of the parents. For example, if the child is anxious, the caregiver may choose to encourage the child to talk about his/her feelings (i.e., active strategy) and/or try to distract the child from their worries (i.e., avoidant strategy), and/or to provide extra information (i.e., information-focused strategy). To conclude, parent–child emotion regulation strategies are found to be related with the child’s mental health, but further research is needed to investigate the direction of relationships before we can advise caregivers in their decisions about communicating with their young children.

For the third factor investigated, higher levels of caregiver’s distress (i.e., caregiver’s mental health and feelings of rejection towards their child) showed to be associated with more mental health problems in the children. This is in line with previous findings showing that mental health of caregivers is directly related with the child’s mental health, also during COVID-19 pandemic [19, 29, 51]. More feelings of rejection of caregivers towards their child were consistently associated with more child’s mental health problems in all six child outcomes. For the interpretation of the results regarding ‘feelings of rejection’, it is important to note that the term ‘rejection’ may be a strong term given the content of (some of) the items of the scale—such as ‘At times the demands that my child makes feel like a burden’- and the relatively low mean scores of the current sample. Therefore, the scale of ‘feelings of rejection’ may be conceptually closer to ‘parental burden’. Both parental burden and parental rejection have previously been associated with more mental health problems in children [26, 52], which can be explained by the parent signaling negative thoughts or feelings towards the child, or being less able to meet the child’s need impacting children’s well-being. However, the opposite direction, in which parental burden was found to be a result of children’s mental health difficulties, has also been reported [53]. Furthermore, maternal rejection, as well as parental burden, have been found to play a mediating role in the relationship between children’s and parent’s mental health [52, 54]. Future research using a longitudinal design could examine the direction of the effect as well as potential mediation mechanisms.

Several limitations warrant caution in the interpretation of the results. First, our study spanned a period of different COVID-19 restriction measures including the closure and re-opening of day cares and schools. We explored whether the scores on the measured constructs differed between the participants that answered the questionnaire during the most strict measures (i.e., closure of day cares and primary schools) compared to the less strict measures (i.e., (re-)opening of day cares and schools). As we found no differences in responses between these two groups on the measured constructs, we think that the impact of the exact restrictions at the time of filling out the questionnaire was limited. Second, relying exclusively on parent-reported questionnaires enabled us to include a large sample, but it also increased the risk of method bias and therefore associations might be overestimated. For example, caregiver’s feelings of general stress and internalizing symptoms as well as an experience of greater negative impact by the pandemic may affect the way in which caregivers perceive their children, increasing the risk of over reporting child mental health problems [55]. Furthermore, most participants in this study had a high socioeconomic status (SES). It is therefore unclear whether the results of the current study also apply to people with a lower SES. As previous research showed that the impact of the COVID-19 pandemic was worse for individuals with low SES [56, 57], it is important for future research to try to include this group specifically in studying the impact of a crisis. Last, as noted, the cross-sectional design of the study withholds us from drawing firm conclusions about the direction of effects between several protective and risk factors in the environment and children’s mental health. As multiple theories and empirical evidence illustrate the transactional nature of the relation between children and caregiver and child-driven effects, for both internalizing as externalizing problems [58–60], an important direction for future research is to understand these family processes during the COVID-19 pandemic.

For clinical use of our findings the following is important. In order to protect children’s mental health during a crisis or other stressful (external) circumstances, specific issues to target seem to be the parental mental health, parents’ subjective experiences of the impact of the pandemic or stressor on daily life and their behavior and communication towards their children about the pandemic or stressor. Psycho-education to inform parents that their perception of a crisis might affect the child’s mental health, may nudge parents towards a less explicit negative perception. Furthermore, screening procedures for parental well-being may be beneficial, in which the parents with mental health problems can enter an intervention targeting their well-being. In the context of a crisis in which social restrictions apply, e-health interventions may be most feasible and have shown to be effective in targeting mental health problems in adults [61].

## Conclusion

To conclude, direct COVID-19 exposure was not related with more mental health problems in children. Family related COVID-19 factors and caregiver’s distress on the other hand were consistently related with more mental health problems and a lower well-being of their young children. In order to protect children’s mental health during stressful circumstances, such as a global crisis, it is advised to direct interventions towards supporting parental mental health, parental perception of the stressful event, and parental communication with their child about the stressor.

### Supplementary Information

**Additional file 1: Table S1.** Descriptive statistics for and correlations among all study variables.

## Data Availability

The datasets used and/or analysed during the current study are available from the corresponding author on reasonable request.

## Abbreviations

PROMIS EC: Patient-Reported Outcomes Measurement Information System Early Childhood
PELQ: COVID-19 Pandemic Exposure and Loss Questions
PIC-EC: Pandemic Impact Questionnaire: Early Childhood
CPPS: COVID-19 Pandemic Parenting Survey
DASS-21: Depression, Anxiety and Stress Scale
PSCQ-T: Parenting as Social Construct Questionnaire Toddler version
