# Supplementary material for: Factors associated with mental health of young children during the COVID-19 pandemic in the Netherlands
Source: Child Adolesc Psychiatry Ment Health. 2023 Dec 13;17:136. doi: 10.1186/s13034-023-00686-9 (PMC10720157; doi:10.1186/s13034-023-00686-9)
Supplement: Supplementary file 1 — Additional file 1: Table S1. Descriptive statistics for and correlations among all study variables. [file 13034_2023_686_MOESM1_ESM.docx]

Additional file 1: Table S1. Descriptive statistics for and correlations among all study variables

|  | 1 | 2 | 3 | 4 | 5 | 6 | 7 | 8 | 9 | 10 | 11 | 12 | 13 | 14 | *N* | *M* | *SD* |
| --- | --- | --- | --- | --- | --- | --- | --- | --- | --- | --- | --- | --- | --- | --- | --- | --- | --- |
| *Children’s Mental Health Outcomes* | | |  |  |  |  |  |  |  |  |  |  |  |  |  |  |  |
| 1. Anger |  |  |  |  |  |  |  |  |  |  |  |  |  |  | 2762 | 7.50 | 2.97 |
| 1. Anxiety | .47^**^ |  |  |  |  |  |  |  |  |  |  |  |  |  | 2637 | 11.56 | 4.39 |
| 1. Depressive symptoms | .50^**^ | .63^**^ | - |  |  |  |  |  |  |  |  |  |  |  | 2508 | 5.73 | 2.27 |
| 1. Sleep problems | .31^**^ | .43^**^ | .33^**^ | - |  |  |  |  |  |  |  |  |  |  | 2315 | 7.72 | 3.15 |
| 1. Positive affect | -.47^**^ | -.43^**^ | -.51^**^ | -.27^**^ | - |  |  |  |  |  |  |  |  |  | 2415 | 16.44 | 1.86 |
| 1. Self-regulation | -.62^**^ | -.39^**^ | -.41^**^ | -.30^**^ | .53^**^ | - |  |  |  |  |  |  |  |  | 2413 | 19.58 | 4.12 |
| *Direct COVID-19 exposure* | |  |  |  |  |  |  |  |  |  |  |  |  |  |  |  |  |
| 1. COVID-19 infection^a^ | -.04^*^ | <-.01 | -.02 | .02 | -.01 | .01 | - |  |  |  |  |  |  |  | 2762 | Yes:  *N* = 317 (11.5%) | |
| 1. Death due to COVID-19^b^ | .03 | .05^*^ | .03 | .05^*^ | .02 | -.02 | .09 | - |  |  |  |  |  |  | 2762 | Yes:  *N* = 152 (5.5%) | |
| *Family related COVID-19 factors* | |  |  |  |  |  |  |  |  |  |  |  |  |  |  |  |  |
| 1. Negative COVID impact | .39^**^ | .41^**^ | .42^**^ | .27^**^ | -.39^**^ | -.38^**^ | .01 | .09^**^ |  |  |  |  |  |  | 2762 | 30.75 | 8.66 |
| 1. Positive COVID impact | -.17^**^ | -.09^**^ | -.12^**^ | -.11^**^ | .24^**^ | .23^**^ | -.02 | .04^*^ | -.19^**^ | - |  |  |  |  | 2762 | 17.88 | 5.02 |
| 1. Avoidant emotion regulation | .16^**^ | .21^**^ | .20^**^ | .09^**^ | -.16^**^ | -.11^**^ | .01 | ..04^*^ | .25^**^ | -.05^*^ |  |  | - |  | 2068 | 6.02 | 2.30 |
| 1. Active emotion regulation | .15^**^ | .18^**^ | .14^**^ | .08^**^ | -.04 | -.03 | .01 | .04^*^ | .11^**^ | .10^**^ | .22^**^ |  |  |  | 2067 | 20.13 | 3.11 |
| 1. Information-focused emotion regulation | .11^**^ | .18^**^ | .12^**^ | .04 | -.09^**^ | -.03 | .02 | .04^*^ | .11^**^ | .11^**^ | .05^*^ | .26^**^ |  |  | 2066 | 1.99 | 1.12 |
| *Caregiver’s distress* |  |  |  |  |  |  |  |  |  |  |  |  |  |  |  |  |  |
| 1. Caregiver’s mental health problems | .35^**^ | .35^**^ | .39^**^ | .26^**^ | -.33^**^ | -.37^**^ | -.01 | .03 | .57^**^ | -.22^**^ | .20^**^ | .03 | .03 |  | 1950 | 19.86 | 18.07 |
| 1. Feelings of rejection | .41^**^ | .28^**^ | .34^**^ | .20^**^ | -.43^**^ | -.47^**^ | -.01 | .02 | .39^**^ | -.20^***^ | .15^**^ | -.01 | .04 | .48^***^ | 2003 | 7.05 | 2.22 |

Note. Pearson correlation are displayed. Point biserial correlations are presented among continuous and categorical variables, and Phi correlations among categorial variables.

^a^COVID-19 infection of parent; 0 = no, 1 = yes
^b^Death of family member or close friend due to COVID-19; 0 = no, 1 = yes
